# Camera Traps Document Infant Corpse Carrying Behaviour in Multiple Unhabituated Chimpanzee Populations

**DOI:** 10.1002/ece3.71698

**Published:** 2025-07-04

**Authors:** Elena Bersacola, Matthew R. McLennan, Joana H. Bessa, Henry Camara, Maimuna Jaló, Vicent Kiiza, Gnan Mamy, Nicholas Mpanga, Vicky Oelze, Marina Ramon, Américo N. W. Sanhá, Laura van Holstein, Fiona A. Stewart, Maegan Fitzgerald, Kathelijne Koops, Kimberley J. Hockings

**Affiliations:** ^1^ Centre for Ecology and Conservation University of Exeter Penryn UK; ^2^ Bulindi Chimpanzee and Community Project Hoima Uganda; ^3^ Faculty of Humanities and Social Sciences Oxford Brookes University Oxford UK; ^4^ School of Life and Health Sciences Roehampton University London UK; ^5^ Institute de Recherche Environnementale de Bossou Bossou Guinea; ^6^ Instituto da Biodiversidade e das Áreas Protegidas Bissau Guinea‐Bissau; ^7^ Anthropology Department University of California Santa Cruz Santa Cruz California USA; ^8^ Clare College University of Cambridge Cambridge UK; ^9^ Department of Anthropology University College London London UK; ^10^ Department of Human Origins Max Planck Institute for Evolutionary Anthropology Leipzig Germany; ^11^ Ape Behaviour and Ecology Group, Department of Evolutionary Anthropology University of Zurich Zurich Switzerland

**Keywords:** dead infant carrying, great ape behaviour, passive wildlife monitoring, remote behavioural monitoring, thanatology, wildlife health

## Abstract

Camera traps are an important tool for wildlife research, particularly for estimating species distribution and habitat use. Although they are increasingly used to study animal behaviour, such as tool use and foraging, there are fewer examples of their use in detecting rare behaviours that cannot be predicted in terms of where and when they may occur. Comparative thanatology, the study of non‐human animal responses to death, has demonstrated that examining behaviours such as infant corpse carrying (ICC) can offer valuable insights into animal cognition, including maternal bonds, grief, and levels of death awareness. Here, we investigate the efficacy of camera traps in capturing ICC in four unhabituated chimpanzee ( *Pan troglodytes* ) populations across West and East Africa, involving a total of 18 chimpanzee communities. We compare ICC detection rates and associated demographic and behavioural data derived from camera traps to published cases recorded through direct observations of habituated communities, the only previous source of ICC reports in wild chimpanzees. Camera traps recorded ICC in seven communities at an average rate of 0.46 cases/year, 2.3 times higher than the 0.20 cases/year recorded through direct observations in 10 habituated communities. The carrying duration in the 10 ICC cases recorded by camera traps ranged from a day or less to at least 28 days (median = 7 days). All 10 ICC cases involved deceased infants with an estimated age bracket between 0 and 0.5 and 2 and 3 years (median: 1 and 1.5 years), and eight out of 10 cases involved a single adult female carrier. Associated demographic and behavioural data support predictions around mother–infant bonds, post‐parturient conditions, and death awareness hypotheses. We conclude that ICC is more common than previously reported in chimpanzees and that camera traps can effectively capture infrequent behaviours such as ICC, making them a promising non‐invasive tool for studying animal behaviour across large spatial scales.

## Graphical abstract

We demonstrate the efficacy of camera traps in capturing infant corpse carrying (ICC) in four chimpanzee populations across West and East Africa. Using camera trap data from seven wild, unhabituated chimpanzee communities, we estimate that average yearly ICC rates are over two times higher than those from published records of habituated communities and demonstrate the potential of camera traps for testing hypotheses concerning ICC—including maternal bond strength, maternal reproductive stage, environmental conditions, and death awareness—at a broader scale. Thus, the wide applicability of camera traps offers the potential to enhance our understanding of primate thanatology and the evolutionary underpinnings of animal cognition and emotions related to death.

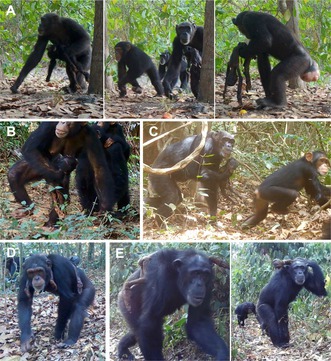

## 1 Introduction

Remote monitoring techniques, such as motion‐triggered camera trapping and passive acoustic recording, are increasingly employed to study animal behaviour in the wild (Caravaggi et al. 2017; Oestreich et al. 2024). Such methods are particularly important in the face of ongoing environmental change, as they enable research on wide spatial scales and are minimally invasive. Camera traps have been successfully used to examine various aspects of wildlife behaviour, including group composition and social relationships (McCarthy et al. 2019; van Leeuwen et al. 2020; Debetencourt et al. 2023; Twining et al. 2024), tool use and behavioural diversity (Kühl et al. 2016; Boesch et al. 2017; Kalan et al. 2019, 2020; Bessa et al. 2022; Bowland et al. 2025), interspecific interactions (Head et al. 2012; Triguero‐Ocaña et al. 2020; Gelmi‐Candusso et al. 2023), foraging (Koops et al. 2019; Johnson et al. 2020) and behavioural responses in human‐modified environments (Ancrenaz et al. 2014; Krief et al. 2014; Smit et al. 2019; Bersacola, Hill, and Hockings 2021; Lee et al. 2024).

Camera traps can also be combined with experimental methods to study specific behaviours by providing visual, auditory, or olfactory cues, or objects in front of the cameras, to increase the chances of recording a behaviour of interest. For example, this approach has been used to study predator–prey interactions (Smith et al. 2020) and tool use (Koops et al. 2022; Luncz et al. 2024). However, not all behaviours can be induced using experiments, and infrequent, unpredictable behaviours may be difficult to detect unless a large amount of data are available. Large datasets can require a considerable amount of time and effort to analyse. Ongoing developments in artificial intelligence (AI) technology are improving its efficiency in automating the detection of species, individuals, and behaviours from camera trap footage, helping to process large datasets (Bain et al. 2021; Vélez et al. 2023). Despite AI advancements, it remains necessary to assess the effectiveness of remote data collection methods, such as camera traps, in detecting specific and/or rare behaviours, especially those that are difficult to predict or experimentally induce, before they can be integrated into a standardised methodological framework. Such assessments remain scarce, despite the widespread use of camera traps in monitoring terrestrial wildlife.

### 1.1 Infant Corpse Carrying: A Rare Yet Revealing Behaviour in Animal Cognition

Some fields of research rely upon drawing together observations of rare or infrequent behaviours across multiple animal groups, populations, and species. For example, comparative thanatology (the interdisciplinary study of death and dying in non‐human animals) examines behaviour around dead and dying conspecifics and heterospecifics and the social and environmental context in which the behaviour occurs (Piel and Stewart 2015; Anderson 2016; Gonçalves and Biro 2018). A variety of behaviours towards dead conspecifics have been recorded across the animal kingdom, with some highly social mammals in particular showing grief‐like responses, such as keeping vigil and guarding the dead body, social withdrawal, increased stress levels, and emaciation (Reggente et al. 2016, 2018; Gonçalves and Biro 2018; Gonçalves and Carvalho 2019; Goldenberg and Wittemyer 2020). Among non‐human primates (hereafter ‘primates’), data from direct observations of habituated individuals have also shown compassionate behaviours towards bereaved individuals, such as increased care towards the mother of a deceased infant by other members of the social group (Anderson 2017; Gonçalves and Carvalho 2019; Goldsborough et al. 2020).

Infant corpse carrying (ICC, i.e., the behaviour of carrying a deceased infant, usually involving the mother carrying the body of her deceased offspring; Watson and Matsuzawa 2018) is the most widely reported behaviour associated with the death of an infant and is performed by many animal species, particularly among cetaceans (Bearzi et al. 2018) and primates (Fernández‐Fueyo et al. 2021). To date, primates are the only taxa recorded to carry infant carcasses for extended periods, i.e., 10 days or longer (Fashing et al. 2011, but see the first recorded extended case in orcas: Cuthbert and Main 2018). Studies of primate thanatology have increased in recent years, with scientists seeking to accumulate anecdotal and sparse evidence from direct observations to conduct quantitative analyses and understand the evolutionary underpinnings of animal cognition and emotions related to death and dying (Anderson et al. 2018; Fernández‐Fueyo et al. 2021; http://thanatobase.mystrikingly.com/).

ICC has so far been observed in 40 non‐human primate species, all of which are haplorrhines, with great apes and catarrhine monkeys documented as displaying prolonged carrying behaviour (Fernández‐Fueyo et al. 2021). Great apes exhibit ‘high order’ cognitive abilities, including theory of mind (Krupenye et al. 2016) and empathy (Clay and de Waal 2013), and it has been argued that they are among those species most likely to possess emergent properties of a human‐like death concept (Gonçalves and Carvalho 2019; De Marco et al. 2022). Behavioural observations of habituated chimpanzees ( *Pan troglodytes* ) across multiple long‐term study communities suggest that while ICC is not uncommon in this species, its incidence is relatively low (Biro et al. 2010; Fashing et al. 2011; Hanamura et al. 2015; Lonsdorf, Wilson, et al. 2020; Botting and van de Waal 2020; Soldati et al. 2022). Opportunities for ICC are rare, however, as it requires the death of an infant, which occurs relatively infrequently in slowly reproducing species such as chimpanzees.

### 1.2 Leading Hypotheses and Predictors for ICC

Several hypotheses to explain the factors influencing mothers' responses to their infants' deaths are being considered in primate thanatology (Sugiyama et al. 2009; Watson and Matsuzawa 2018; Das et al. 2019; Lonsdorf, Wilson, et al. 2020; Fernández‐Fueyo et al. 2021). First, it is hypothesised that newborns might be carried for longer due to the extended delay in the mother ceasing lactation and restarting their reproductive cycle after giving birth (‘post‐parturient condition hypothesis’, e.g., Kaplan 1973). Alternatively, extended carrying might be constrained by energetic costs, particularly in smaller species where the adult‐infant body size ratio is smaller (Fernández‐Fueyo et al. 2021).

Another hypothesis suggests that mothers develop a strong bond with infants older than a few days or weeks that could continue after the infant's death (‘mother–infant bond hypothesis’, e.g., Kaplan 1973; Sugiyama et al. 2009; Li et al. 2012; Watson and Matsuzawa 2018; Fernández‐Fueyo et al. 2021). A cross‐species comparative study of ICC in primates showed a quadratic relationship, with intermediate‐aged infants carried for longer, supporting the ‘mother–infant bond hypothesis’ with the notion that bonding peaks over time and decreases just before weaning (Fernández‐Fueyo et al. 2021).

Sensory cues may be important for mothers to become aware of their offspring's death (‘death awareness hypothesis’) and to process the premature separation from them (Cronin et al. 2011). Using ICC data on 40 primate species, researchers found that younger mothers were more likely to carry their deceased offspring and that ICC was more likely to occur when the death of the infant was non‐traumatic (Fernández‐Fueyo et al. 2021). These findings support the hypotheses that younger mothers may be less aware of death compared to experienced mothers and that contextual cues, such as a violent death, may increase death awareness, thus influencing whether or not ICC is performed. The ability to distinguish between dead and alive has been termed ‘associative concepts’ in death awareness research (Gonçalves and Carvalho 2019). Aside from the mother's age (and experience with death) and the cause of the infant's death, atypical carrying modes such as slinging corpses over the shoulders, dragging them on the ground, or carrying them by their head have also been associated with the awareness of the difference between ‘dead’ and ‘alive’, i.e., the carrier may be aware the corpse cannot feel pain or discomfort and may adjust their carrying mode accordingly (e.g., Das et al. 2019; Lonsdorf, Wilson, et al. 2020).

In addition to the above hypotheses, it has also been suggested that ICC duration may be influenced by climatic conditions impacting the rate of carcass decomposition (‘slow decomposition hypothesis’, e.g., Biro et al. 2010; Fashing et al. 2011). It was hypothesised that extended ICC is more likely to occur during dry hot seasons, when the process of putrefaction is slowed through desiccation and mummification (Fashing et al. 2011; Piombino‐Mascali and Carr 2020). However, this hypothesis has since been refuted using data from 40 primate species (Fernández‐Fueyo et al. 2021) and among chimpanzee ICC data specifically (Lonsdorf, Wilson, et al. 2020). Using 30 chimpanzee ICC cases, Lonsdorf, Wilson, et al. (2020) also found no statistical support for the post‐parturient condition, mother–infant bond, and death awareness through maternal experience hypotheses. Further cross‐site comparisons involving a broader range of environmental contexts and more chimpanzee communities could provide additional insights into the factors influencing this behaviour.

### 1.3 Expanding Data Collection Methods in Thanatology: The Role of Camera Traps

ICC has been observed in habituated chimpanzee communities of eastern chimpanzees (*P. t. schweinfurthii*) and western chimpanzees (*P. t. verus*) at six long‐term study sites but has not been reported in the central (*P. t. troglodytes*) and Nigeria‐Cameroon subspecies (*P. t. ellioti*), likely due to study bias. Most chimpanzee communities remain unhabituated to human observers across their geographic range, raising questions about the potential of indirect methods, such as camera traps, for documenting ICC. Indeed, for great apes, behavioural data can be obtained without the time‐consuming and costly process of habituation required for observational research (e.g., Bessa et al. 2022; Koops et al. 2022). Camera traps also minimise negative impacts on animals, such as the risk of disease transmission, hunting, and behavioural alteration due to the researcher's presence (Hansen et al. 2022; Mitani et al. 2024). Camera traps have successfully detected rare or infrequent chimpanzee behaviours, such as nocturnality and tool use (Boyer‐Ontl and Pruetz 2014; Krief et al. 2014; Lapuente et al. 2017; Tagg et al. 2018; Bessa et al. 2021; Lacroux et al. 2022). Fewer examples exist for their use in capturing rare behaviours that leave no trace or do not predictably occur in a known location (i.e., where targeted camera traps can be set up). It remains unknown whether camera traps can reliably capture ICC cases and how data from camera traps compare to behavioural observations of ICC from habituated communities.

The primary aim of this study is to investigate the efficacy of camera traps in capturing the occurrence of ICC. To achieve this, we provide data on the presence and annual rates of ICC cases in 18 unhabituated chimpanzee communities, recorded by camera traps across four research sites in West and East Africa. We compare these with published data from direct observations of habituated chimpanzees at six long‐term research sites. We extract demographic and behavioural information to evaluate the effectiveness of camera trap footage for testing hypotheses related to the drivers of ICC occurrence and duration, including predictors such as maternal reproductive stage, infant age, carrying modes, and environmental conditions (Gonçalves and Carvalho 2019; Lonsdorf, Wilson, et al. 2020). Finally, we discuss the benefits and limitations of relying on camera traps for studying rare wildlife behaviours such as ICC.

## 2 Materials and Methods

### 2.1 Study Species

Chimpanzees range across West, Central, and East Africa; from Senegal and Guinea‐Bissau in the northwest to South Sudan, Uganda, and Tanzania in the East (Humle et al. 2016, Figure 1). Their range comprises different ecoregions, climates, and habitat types, from dry arid savannahs to semi‐deciduous coastal forests, tropical rainforests, and montane forests (Humle et al. 2016). Anthropogenic influence varies across gradients (Hockings et al. 2015), from remote rainforests (e.g., Morgan and Sanz 2003) to human‐dominated landscapes where the dominant land use is agriculture, with little forest cover remaining (McLennan et al. 2021). Chimpanzees are present extensively outside protected areas, particularly in forest‐savannah‐agriculture mosaics and where hunting is low or absent (McCarthy et al. 2015; Heinicke, Mundry, Boesch, et al. 2019; Heinicke, Mundry, Boesch, Amarasekaran, et al. 2019; Estrada et al. 2022).

**FIGURE 1:**
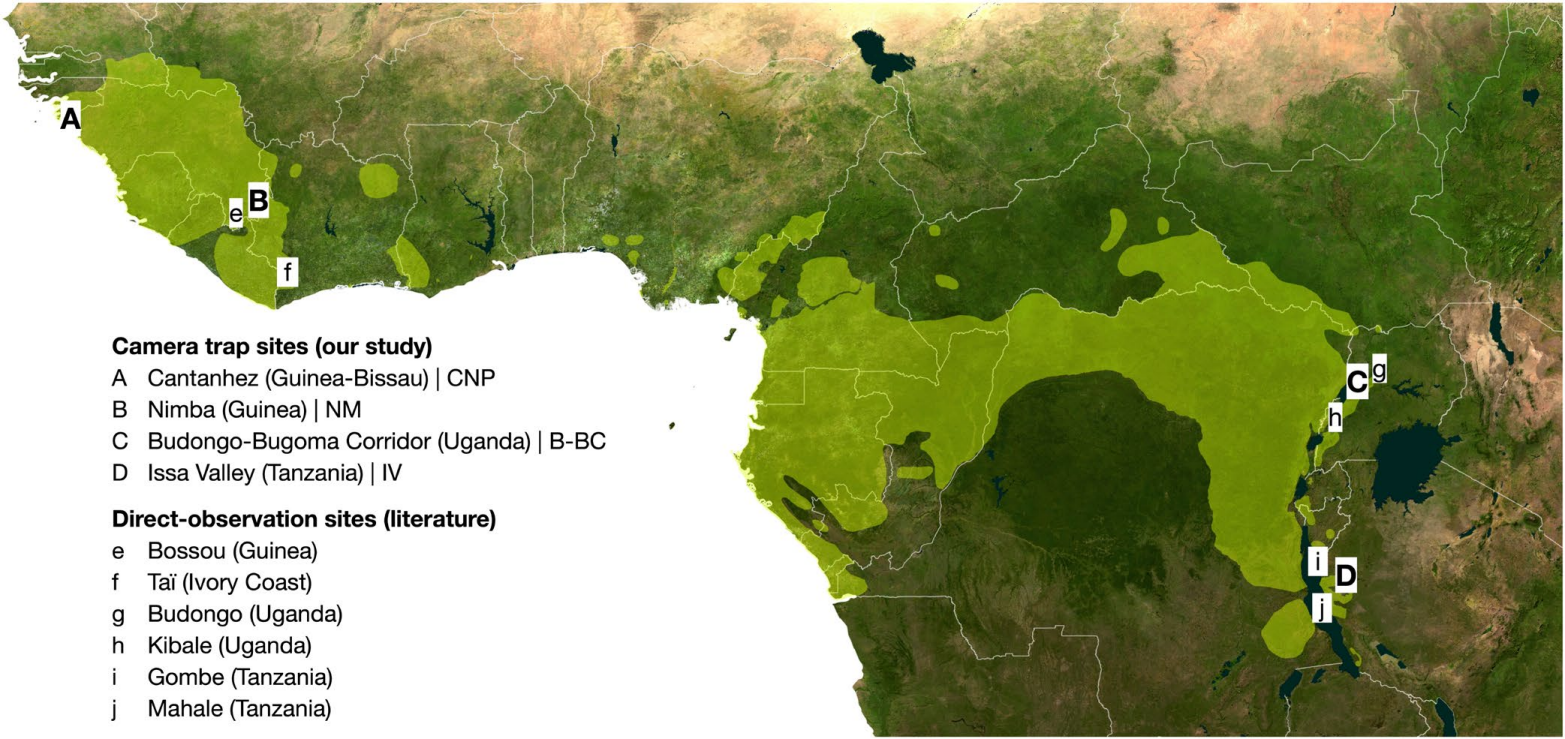
Chimpanzee study sites across Africa where ICC behaviour is reported to occur based on camera trap monitoring (our study) and direct observations of habituated individuals (published literature). Light green represents chimpanzees' geographical range (Humle et al. 2016). Contains satellite imagery (NASA Worldview).

Chimpanzees live in hierarchical groups, known as ‘communities’, typically ranging from about 10 to 120 individuals (Sugiyama 2004; Vieira et al. 2019; Badihi et al. 2022), with the largest recorded community comprising over 200 individuals (Langergraber et al. 2017). Chimpanzees display fission‐fusion dynamics, where the community contains smaller social groups, known as ‘parties’, that split (fission) and merge (fusion), changing in response to local socio‐ecological conditions (Nishida 1968; Goodall 1986; Lehmann and Boesch 2004; Hockings et al. 2012). Home range size varies depending on the habitat and availability and distribution of resources, typically ranging from approximately 7–60 km^2^ (Edwards et al. 2023).

### 2.2 Study Areas

We present data from four chimpanzee research sites, including western chimpanzees (*P. t. verus*) at Cantanhez National Park (CNP) in Guinea‐Bissau, the Nimba Mountains (NM) in Guinea, eastern chimpanzees (*P. t. schweinfurthii*) at the Budongo‐Bugoma corridor (B‐BC) in Uganda, and Issa Valley (IV) in Tanzania (Table 1).

**TABLE 1:** Landscape characteristics, chimpanzee community size (including the number of adult females, if known), estimated home range size, and sampling effort across the 18 chimpanzee communities in West and East Africa.

Site	Landscape type, habitats	Chimpanzee community	Community size	Study period	No of years sampled	No of months sampled	Number of camera trap clusters	Camera deployment type (min distance in km)	Study area size (km^2^; s100% MCP of CTs)	Estimated home range size (km^2^; 100% MCP)
CNP	Protected Landscape. Mosaic of coastal forest blocks, mangroves, regenerating forest patches, savannah and human land use (agriculture, villages, roads)	Caiquene–Cadique	≥ 49 (≥ 17 adult females)^b^	2015–2023	9	68	84	Targeted (0, 0.5); General (1)	12.8	14.9
		Lautchande	Unknown	2015–2022	7	42	47	Targeted (0); General (1)	10.9	11.8
		Cambeque	Unknown	2016–2022	6	38	47	Targeted (0); General (1)	16.3	17.9
		Madina	Unknown	2016–2022	6	38	36	Targeted (0); General (1)	14.6	21
		Caghode–Cabante	Unknown	2017–2022	6	36	21	Targeted (0); General (1)	9	13.8
		Canamine	Unknown	2017–2022	6	24	13	General (1)	12.3	~16
		Amindara	Unknown	2017–2022	5	27	18	General (1)	21.6	~28
		Guiledje^a^	Unknown	2018–2022	4	23	16	Targeted (0); General (1)	12.2	Unknown
		Faro Sadjuma^a^	Unknown	2020–2022	3	19	≥ 2	General (1)	na	Unknown
		Gandembel–Balana^a^	Unknown	2020–2022	3	19	8	General (1)	10.1	Unknown
NM	Protected Nature Reserve. Primary tropical forest, savannah grasslands, terrestrial herbaceous vegetation, riverine forest	Gahtoy	47^c^ independent (adults and adolescents) individuals	2022–2023	2	16	22	Targeted (0)	4.6	12.5
		Tongbongbon	42^c^ independent (adults and adolescents) individuals	2022–2023	2	16	21	Targeted (0)	8.6	16.7
B‐BC	Unprotected. Human‐dominated, highly anthropogenic, mostly agriculture and timber plantations with fragments of degraded riverine forest	Mairirwe	27–30 (9 adult females)	2018	1	4	10	Targeted (0)	0.5	≥ 11.3^d^
		Wagaisa	34–36 (10 adult females)	2019–2021	3	11	9	Targeted (0)	2.8	≥ 42.5^e^
		Kyabasengya	17 (6 adult females)	2019	1	1	2	Targeted (0)	0.1	≥ 10
		Kihomboza	16 (6 adult females)	2022	1	3	4	Targeted (0)	0.1	≥ 25
IV	Protected Reserve since 2017. Miombo woodland, riparian forests, rocky outcrops mosaic	Issa	27 (8 adult females)^e^	2011–2024	13	156	60	Targeted (0.15)	15–58	≥ 39^f^
		Other (non‐Issa)	Unknown	2011–2024	13	156	≥ 1	Targeted (0.15)	Unknown (< 58)	Unknown

*Note:* Number of years and months sampled are calendar years when at least 1 month per year or 1 day per month of camera trap sampling took place, respectively. Number of camera trap ‘clusters’ refers to the total number of independent camera trap locations used, deployed at least 20 m apart. Camera deployment type includes chimpanzee‐targeted studies (targeted) and biomonitoring/occupancy studies of terrestrial mammals (general). The minimum distance between camera trap locations in kilometres is shown in brackets, with zero values indicating no minimum distance requirements. Study area and estimated home range size are derived using 100% minimum convex polygons of camera trap locations and wider location/ranging data for the chimpanzee community, respectively.

^a^ Indicates that the home range of the chimpanzee community is not fully known, and that the area may comprise more than one community.

^b^ Preliminary data based on individual identification from camera trap footage between July and December 2018 (Ramon 2025).

^c^ Based on genetic censusing (Koops et al. 2023).

^d^ Based on (McLennan et al. 2019).

^e^ Based on (McLennan et al. 2021).

^f^ Based on post‐habituation observations (Drummond‐Clarke et al. 2022).

Cantanhez National Park (CNP, 1067 km^2^) constitutes a Protected Landscape, IUCN Category V (IUCN 2016), containing a mosaic of coastal forest patches, mangroves, savannah, and agriculture (Bersacola and Hockings 2023). CNP includes over 25,000 residents across ~200 villages. At least 10 chimpanzee communities comprising c. 500 individuals (Bersacola et al. in prep.) are known to be present in CNP and all are unhabituated (Bersacola, Parathian, et al. 2021; Hockings et al. 2021). Chimpanzee research at CNP largely relies on indirect methods of observation such as camera traps (Bersacola, Hill, and Hockings 2021; Bersacola et al. 2022; Bain et al. 2021; Bessa et al. 2022). The climate is characterised by one monsoon‐like rainy season (June–November) and a long dry season with no rainfall (December–May). Total annual rainfall averaged 2626 mm in 2010–2021 (Harris et al. 2020). Temperature fluctuations are higher during the dry season (min/max 17°C–38°C in 2010–2021, Harris et al. 2020) compared to the rainy season (22°C–34°C).

The Seringbara study site is located within the Mount Nimba Strict Nature Reserve in the Nimba Mountains of Guinea, West Africa. The study site spans an area of c. 30 km^2^ and the chimpanzees have been the subjects of research since 2003 (Koops 2011). Due to the challenging terrain, the chimpanzees remain largely unhabituated to the presence of researchers. Camera traps have been employed at the study site since 2007 (Koops et al. 2022). The study site has great topographic diversity with altitudes ranging from 600 m in the deepest valley to over 1750 m at the highest peak (Koops 2011). Vegetation comprises primary tropical forest, with areas of savannah grassland, terrestrial herbaceous vegetation, and riverine forest (Koops et al. 2012). Two chimpanzee communities reside in the Seringbara research area: the Gahtoy and Tongbongbon communities (Koops et al. 2023). Based on genetic censusing, the Gahtoy community has an estimated total of 47 independent (i.e., adolescents, adults) chimpanzees, and the Tongbongbon community an estimated total of 42 independent individuals (Koops et al. 2023). The climate is characterised by an extended rainy season (March–November, > 60 mm rainfall monthly), followed by a 3‐month dry season (≤ 60 mm rainfall monthly, December–February; Koops 2011; Koops et al. 2013). Total annual rainfall averaged 2024 mm in 2010–2021 (Harris et al. 2020). Minimum and maximum temperatures normally occur in the dry season (min/max 12.7°C–35.8°C in 2010–2021, Harris et al. 2020).

The Budongo‐Bugoma Corridor (B‐BC) consists of an unprotected, human‐dominated landscape covering c. 1500 km^2^ between the Budongo and Bugoma Central Forest Reserves in western Uganda. About 300 chimpanzees in 10 or more small communities survive amidst a high‐density human population, using remnant patches of degraded riparian forest, agricultural land and exotic timber plantations around villages and urban centres (McLennan 2008; McCarthy et al. 2015; McLennan et al. 2021). While one chimpanzee community (Bulindi) is habituated, having been studied since 2006 (Satsias et al. 2022), camera traps have been used to obtain demographic data in four other unhabituated communities in the B‐BC (McLennan et al. 2019, 2021). Rainfall in the region is bimodal. March–May and August–November are usually wet (> 100 mm rainfall monthly), December–February are dry (≤ 50 mm rainfall monthly), while June–July are ‘transient’ (51–100 mm rainfall; McLennan et al. 2017). Total annual rainfall averaged 1350 mm in 2010–2021 (Harris et al. 2020). Minimum and maximum temperatures normally occur in the January–March dry season (16°C–32°C, 2010–2021, Harris et al. 2020).

The Issa Valley (IV) is situated within the Tongwe West Forest Reserve within the Greater Mahale Ecosystem (GME) in western Tanzania, more than 90 km from the nearest national park boundary (Mahale Mountains National Park) on Lake Tanganyika (Piel et al. 2019). Tongwe West Forest Reserve was gazetted in 2017, and prior to this, it was unprotected general land. The wider GME covers approximately 18,000 km^2^ and consists of a mosaic habitat characterised as a miombo woodland (dominated by *Brachystegia* and *Julbernardia* species), with interspersed riparian forest strips and rocky outcrops. The Issa chimpanzee community numbers 27 individuals and is situated within the wider population of approximately 3800 chimpanzees despite average annual declines of > 2% of the GME's chimpanzees in the last 10 years (Carvalho et al. 2022). At IV, chimpanzees have been studied using remote monitoring (acoustics, camera traps) since 2008 (Stewart and Piel 2014; Moore et al. 2017; Piel 2018). The Issa community shares part of its range with at least one neighbouring community, and individuals are identified on camera trap footage as Issa community or non‐Issa community members. The climate is characterised by a single 6‐month wet season from November to April and a dry season spanning May–October, with the total annual rainfall averaging 1198 mm in 2010–2021 (Harris et al. 2020). Minimum temperatures occur in June–July during the dry season (11.5°C in 2010–2021, Harris et al. 2020); maximum temperatures occur in August–October (dry‐wet season transition, 28.5°C).

### 2.3 Camera Trap Data Collection

#### 2.3.1 Cantanhez National Park, Guinea‐Bissau

In CNP, camera trap research included chimpanzee‐targeted research and systematic biomonitoring of medium‐to‐large terrestrial mammals. Chimpanzee‐targeting camera traps focused on one to six chimpanzee communities depending on the study (e.g., home range use in one community: Bersacola, Hill, and Hockings 2021; cultural variation across four communities: Bessa et al. 2022; leprosy occurrence across six communities; and demography/social structure across three communities: Ramon 2025). Targeted cameras pointed towards chimpanzee paths, freshwater sources, as well as feeding, tool use, and buttress drumming sites (Bessa et al. 2022). Minimum distance between targeted camera traps varied from being not considered (in behavioural studies) to c. 500 m (home range use), with some deployed using a grid‐based approach with one camera for each 1 km^2^ (health and demography/social structure). Chimpanzee‐targeting camera traps were set up in photo (three photos per trigger), video (15–60 s) and hybrid mode with 1–10 s intervals. Biomonitoring research in 2016–2017 covered c. 180 km^2^, which overlapped with the home range of seven chimpanzee communities (Bersacola et al. 2022). In 2020–2022, a systematic biomonitoring program including camera traps was established across CNP, covering 550 km^2^, a research area overlapping with the home range of at least 10 chimpanzee communities (Bersacola, Hill, and Hockings 2021, Cantanhez Chimpanzee Project, unpubl. data). Biomonitoring camera traps were deployed pointing towards animal paths, including those used by chimpanzees, with a minimum spacing between camera traps of 1 km. Biomonitoring camera traps were programmed to record photos (three per trigger) with 5–10 s intervals, though occasional malfunctions reset some of these cameras to video mode. In CNP, we used Bushnell (Bushnell Trophy Cam models) and Browning (Recon Force and Spec Ops models) camera traps, positioned at heights of 0.6–1 m. Between 2015 and 2023, we established 270 chimpanzee‐targeted camera traps and 128 biomonitoring camera traps. As some of these camera trap locations overlapped across the years, we calculated the number of independent locations (camera trap clusters) using 20 m as the maximum distance between camera traps in each cluster, yielding a total of 309 camera trap clusters used across CNP between September 2015 and March 2023. As of March 2023, total camera trap sampling effort in CNP over the 9‐year study period was approximately 70,000 camera trap days.

#### 2.3.2 Seringbara, Nimba Mountains, Guinea

For this study, camera trapping took place during *N* = 16 months continuously between February 2022 and June 2023 (excluding October 2022). The total number of camera trap clusters deployed in Seringbara was 40. We employed 19 camera trap clusters exclusively in the Gahtoy home range, and 18 exclusively in the Tongbongbon home range. In addition, three camera trap clusters were in an overlap zone and visited by both communities. Camera traps (Browning Trail Cameras Recon Force Elite HP4) were deployed at a height of about 1 m to record 60‐s video with a 1‐s interval. Cameras were placed at chimpanzee trails (van Leeuwen et al. 2020; Koops et al. 2019), at buttress drumming trees (Fitzgerald et al. 2022) and at crab‐fishing sites (Koops et al. 2019; Koops et al. 2024). Camera trap survey effort during the study period was > 13,000 camera trap days.

#### 2.3.3 Budongo‐Bugoma Corridor, Uganda

During 2018–2022, camera traps were deployed at different times in the home ranges of four chimpanzee communities (Mairirwe, Wagaisa, Kyabasengya and Kihomboza). At each site, between two and six targeted cameras (Bushnell Trophy Cam HD Aggressor; Campark T85; Crenova HC802A) were positioned simultaneously on chimpanzee trails within small, dense forest patches or, at Kihomboza, in exotic timber and agroforestry plantations. The primary purpose was to gain demographic information about each community prior to longer‐term conservation monitoring (McLennan et al. 2021). However, at Mairirwe, camera traps were also positioned to document tool use (McLennan et al. 2019). Camera traps were generally deployed for as long as required to identify the members of each chimpanzee community. In the small Kihomboza and Kyabasengya communities (each comprising 16–17 individuals during the period considered here) this was achieved within 1–2 months, whereas ≥ 4 months were required to identify individuals in the larger Mairirwe and Wagaisa communities (27–30 and 34–36 individuals, respectively). In three of the four communities, camera traps were used continuously during a single short period (≤ 4 months; Table 1). However, cameras were re‐deployed three times at Wagaisa after intervals of 2, 15, and 4 months, respectively, to confirm the presence/absence of rarely seen individuals, giving a total deployment period of 11 months at this site. Camera traps were set at a height of about 0.5 m to record 15–90 s (median 60 s) video with a 1–5 s interval between recordings. The total number of independent camera trap locations (i.e., clusters, where 20 m was the maximum distance between cameras in a cluster, as per CNP) was 25 (range 2–10 per community); cameras were at times moved to new locations to increase the likelihood of capturing chimpanzees when the existing location yielded unsatisfactory results. Camera trap survey effort (all communities combined) totalled 1332 camera trap days.

#### 2.3.4 Issa Valley, Tanzania

Data from camera traps used in the current study span the period before and after habituation of the Issa chimpanzees which occurred in summer 2018 and since then, they have been followed daily (Giuliano et al. 2022). From 2011 to 2019, the research team managed an array that began with ~40 motion‐triggered cameras (Bushnell Trophy Cam, Browning Strike Force, and Reconyx Ultrafire) across 18 km^2^, before expanding in number (> 50 cameras, 46 km^2^) in 2020 to the current array of 60 camera traps over 58 km^2^ covered. Cameras have always been deployed non‐randomly pointing towards wildlife trails and termite mounds and representing different vegetation types found in Issa's mosaic habitat (D'Ammando et al. 2022). Camera traps were deployed at least 0.15 km distance from each other at a height of 1 m to record 60‐s video with a 1‐s interval. Camera trap survey effort has varied from 7000 to 10,000 camera trap days per year, totalling > 100,000 camera trap days over the 13‐year study period.

### 2.4 Literature Search for Documented Cases in Habituated Chimpanzee Populations

We searched the published literature for documented cases of ICC in habituated chimpanzee populations. Using Google Scholar, we searched with the keywords “infant corpse carrying” OR “dead infant carrying” AND “chimpanzee” OR “ *Pan troglodytes* ”. Additionally, we cross‐checked ICC reports and retrieved cited literature from published studies that had previously compiled ICC data, such as Fernández‐Fueyo et al. (2021). Our selection criteria required that the chimpanzee communities had been monitored through direct behavioural observations and that they belonged to wild populations, thus excluding cases documented in captive settings.

### 2.5 Data Management and Analysis

We considered an independent ‘event’ as all videos containing chimpanzees recorded at the same location (or camera trap cluster) occurring within 60 min of another (van Leeuwen et al. 2020). We followed Watson and Matsuzawa (2018) to describe ICC and associated behaviours by chimpanzees during independent events, including age and sex of the individual carrying the corpse, mode of carrying and *ad libitum* behaviours. We also estimated the minimum party size, considering fully independent individuals only (adults and subadults/adolescents, e.g., van Leeuwen et al. (2020)), and the demographic composition of chimpanzee parties during independent events containing ICC. Dependants (infants and juveniles) were excluded from party size calculations because we were interested to assess whether ICC carriers—normally adult females (the presumed mothers)—associate with independent individuals (in more or less cohesive parties) or choose to isolate themselves socially. However, any individuals interacting with the corpse, regardless of age category, were reported in the results.

We estimated age categories based on those used at Bossou: infants (0–3 years), juveniles (4–7 years), subadults/adolescents (8–11 years), and adults (≥ 12 years; Sugiyama 2004). We acknowledge that use of these age categories varies among studies, as does, potentially, developmental milestones among sites. The age of dead infants was estimated based on body size and head/body proportions, using visual comparisons with published photographic evidence from long‐term sites where the age of the dead infant was known (Biro et al. 2010; Boesch 2012; Hanamura et al. 2015; Lonsdorf, Wilson, et al. 2020; Soldati et al. 2022), as well as our own extensive experience observing wild chimpanzee infants. Demographic categories, as well as the identities of carriers and corpses, were independently assessed by at least three co‐authors, with consensus reached. We further note here that the authors are experienced at identifying chimpanzee individuals from camera trap footage. Carriers (mostly adult females) that were captured on multiple occasions, including before, during and/or after ICC events, were identifiable to the authors at each field site. We are confident that all cases of ICC reported here involved infants that were deceased and not unconscious. When multiple images or videos captured a fresh infant corpse over time, these cases progressed to decomposition and/or mummification. If only a single image/video of a fresh body was available, additional cues (e.g., dislocated limb, carrying mode) indicated death. Where possible, we reviewed later footage of the adult female carrier to confirm the absence of a live infant.

For each case of ICC, we calculated the minimum ICC duration as the number of days between the first and last independent event recorded on camera traps. We consider an ‘extended’ ICC case when the corpse is carried for longer than 10 days, following Fashing et al. (2011). In ICC cases detected using camera traps, it is often not possible to determine how many days the infant had been dead prior to the first camera trap recording. For example, a seemingly ‘fresh’ corpse could already be a few days old. Therefore, we also consider an ICC case as ‘extended’ when the corpse appears or becomes fully mummified (i.e., all hair lost, with body parts “encased in dry leathery skin”: Biro et al. 2010), assuming the full mummification process takes approximately 10 or more days (Soldati et al. 2022). For ICC cases reported in the literature, we used the duration provided by the authors, rounding up to the nearest integer.

To compare the frequency of ICC detections between camera trap data (from this study) and direct observations (from the literature review), we calculated the number of ICC cases per year of research conducted for each chimpanzee community. We first compared across all communities, including those where we did not record the behaviour, and then included only communities where ICC was recorded at least once. We focused on comparing data from chimpanzee communities where the behaviour was observed by either method, as unreported ICC cases in other studied habituated communities may result from a lack of publication rather than non‐detection.

Due to the limited availability of research effort information (i.e., exact dates and times for direct observations in published data from long‐term study sites were unavailable), we used rounded years (integers) as a measure of sampling effort for each chimpanzee community studied. Specifically, we counted years of research from habituation and the start of regular behavioural follows up to the year of publication of the study reporting ICC cases. For one community, the end year was defined by the group's extinction (Nakamura et al. 2015). All other chimpanzee communities included in this paper continue to be monitored by researchers to date. As for camera trap data, exact number of camera trap days were not always available for each community in our study, as only systematic monitoring studies (e.g., Bersacola et al. 2022) typically require this information. Given this limitation and to maintain consistency, we applied the same temporal resolution to the camera trap data, using number of years of research as a proxy for survey effort to ensure comparability with ICC cases in the published literature. The number of years sampled refers to calendar years in which camera trapping took place for at least 1 month.

### 2.6 Ethical Note

This research involving wild unhabituated chimpanzees was non‐invasive and complied with the ethics guidelines detailed by the Association for the Study of Animal Behaviour (UK) and with the legal requirements of Guinea‐Bissau, Guinea, Uganda, and Tanzania where the research was conducted.

## 3 Results

We recorded 10 cases of ICC in seven of the 18 unhabituated chimpanzee communities included in this study. These included three of the 10 communities in CNP, Guinea‐Bissau; one of the two communities in NM, Guinea; one of the four communities in B‐BC, Uganda; and two of at least two communities in IV, Tanzania (Table 2). Across these 10 detected cases, the number of independent camera trap events recorded per case ranged from 1 to 27, with a median of two independent events per case. The interval between camera trap events ranged from zero (in cases with one camera trap event) to 28 days, with a median of 7 days between first and last camera trap event across ICC cases. Based on an interval of at least 10 days between camera trap events and/or the state of the corpse, at least seven of the 10 recorded ICC cases were classified as extended ICC. We detected six ICC cases in the dry season, three cases during the transition period from dry to rainy season, and one case in the rainy season. Of the extended ICC cases, four occurred during the dry season while three occurred during the dry‐rainy transition period.

**TABLE 2:** Summary of 10 ICC cases detected by camera traps across seven unhabituated communities, including duration period of camera trap detection, season, number of independent events and camera trap clusters in which the ICC case was detected, number of camera trap clusters active during the ICC case within the chimpanzee community, and deployment type.

Site	Community	Case ID	Recorded duration^a^	Season	Condition of the corpse	Date range of camera trap event (s)	No of independent events (detection mode)	No clusters that detected the case (*N* active clusters)^b^	Deployment type during ICC detection
CNP	Caiquene–Cadique	ICC_CC_1	7 days	Transition from dry to rainy season	Fresh to mummified	17/05/2020–23/05/2020	2 (photo)	1 (5)	General monitoring of mammals
		ICC_CC_2	28 days	Dry	Fresh to mummified	10/12/2022–06/01/2023	2 (photo)	2 (26)	Targeted monitoring of chimpanzees
CNP	Caghode–Cabante	ICC_CGH_1	1 day	Dry	Fresh	30/12/2021	1 (video)	1 (9)	General monitoring of mammals
CNP	Lautchande	ICC_LA_1	2 days	Dry	Fresh	14/03/2022–15/03/2022	2 (video)	2 (18)	2 general, 16 targeted monitoring
NM	Tongbongbon	ICC_TB_1	9 days	Dry	Fresh to mummified	01/02/2023–09/02/2023	3 (video)	3 (18)	Targeted monitoring of chimpanzees
		ICC_TB_2	5 days	Dry	Mummified	16/02/2023–20/02/2023	2 (video)	2 (18)	Targeted monitoring of chimpanzees
		ICC_TB_3	15 days	Transition from dry to rainy season	Mummified	24/03/2023–07/04/2023	2 (video)	2 (21)	Targeted monitoring of chimpanzees
B‐BC	Kihomboza	ICC_KB_1	26 days	Dry	Mummified	07/02/2022–04/03/2022	27 (video)	2 (3)	Targeted monitoring of chimpanzees
IV	Issa	ICC_IV_1	1 day	Rainy	Fresh	17/11/2013	2 (video)	1 (40–60)	Targeted monitoring of chimpanzees
IV	Other (non‐Issa)	ICC_IV_2	1 day	Transition from dry to rainy season	Mummified	17/10/2020	1 (video)	1 (unknown)	Targeted monitoring of chimpanzees

^a^ Defined as the number of calendar days between the first and last recorded independent event of the same ICC case.

^b^ Number of active locations refers to the number of camera trap locations/clusters operating within the community home range during the study period when ICC behaviour was recorded.

### 3.1 Comparing Rates of ICC Detection

We retrieved data on ICC occurrence in wild chimpanzees from direct observations of well‐studied, habituated populations reported in eight publications (Table 3). These ICC reports come from six long‐term study sites across West (*N* = 2) and East Africa (*N* = 4) comprising three and seven chimpanzee communities, respectively. For every year of research, we detected ICC cases in our 18 monitored chimpanzee communities at an average rate of 0.18 cases per year (range: 0–1.5 cases per year), which equates to approximately one case every 5.5 years. These average detection rates are comparable to those recorded from direct observations of the 10 habituated chimpanzee communities in which the behaviour was reported (average 0.20 cases per year, range: 0.06–0.54). Our average yearly detection rate of extended ICC cases across the 18 unhabituated communities was 0.16, over three times higher than the rate from direct observations of habituated animals (yearly average extended ICC cases = 0.05). When considering solely the rate of ICC case detection within our study communities where the behaviour was detected (*N* = 7 communities), the yearly detection rates of ICC cases (0.46 cases/year) and extended ICC cases (0.40 cases/year) recorded by camera traps in unhabituated communities were 2.3 times and 7.9 times higher, respectively, compared to those recorded in habituated communities using direct observations. Overall, 70% of all ICC cases detected by our camera traps (*N* = 10) were extended cases (*N* = 7). From the obtained sample from published literature, 22% of all ICC cases recorded by direct observations (*N* = 69) were extended cases (*N* = 15).

**TABLE 3:** Comparison of rates of ICC detection by camera traps with those derived from published observations at long‐term study sites.

Site	Community	Observation method	Research period (*N* of years)	All ICC cases recorded (yearly rate)	Extended ICC cases (yearly rate)	Study reporting ICC case
Bossou, Guinea	Bossou	Behavioural observations, habituated	1976–2010 (34)	3 (0.09)	3 (0.09)	Biro et al. (2010)
Taï, Ivory Coast	East group	Behavioural observations, habituated	2007–2020 (13)	1 (0.08)	0	Fedurek et al. (2020)
Taï, Ivory Coast	North group	Behavioural observations, habituated	1979–2012 (33)	2 (0.06)	0	Boesch and Boesch‐Achermann (2000); Boesch (2012)
Budongo, Uganda	Sonso	Behavioural observations, habituated	1990–2022 (32)	11 (0.34)	2 (0.06)	Soldati et al. (2022)
Budongo, Uganda	Waibira	Behavioural observations, habituated	2011–2022 (11)	2 (0.18)	1 (0.09)	Fedurek et al. (2020); Soldati et al. (2022)
Kibale, Uganda	Ngogo	Behavioural observations, habituated	1995–2020 (25)	3 (0.12)	2 (0.08)	Watts (2020)
Gombe, Tanzania	Kasekela	Behavioural observations, habituated	1966–2020 (54)	29 (0.54)	1 (0.02)	Lonsdorf, Wilson, et al. (2020)
Gombe, Tanzania	Mitumba	Behavioural observations, habituated	1995–2020 (25)	4 (0.16)	1 (0.04)	Lonsdorf, Wilson, et al. (2020)
Mahale, Tanzania	K	Behavioural observations, habituated	1966‐1983^a^ (17)	3 (0.18)	1 (0.06)	Hanamura et al. (2015)
Mahale, Tanzania	M	Behavioural observations, habituated	1968–2015 (47)	11 (0.23)	4 (0.09)	Hanamura et al. (2015)
Cantanhez, Guinea‐Bissau	Caiquene–Cadique	Camera traps, unhabituated	2015–2023 (9)	2 (0.22)	2 (0.22)	This study
Cantanhez, Guinea‐Bissau	Lautchande	Camera traps, unhabituated	2015–2022 (7)	1 (0.14)	0	This study
Cantanhez, Guinea‐Bissau	Caghode–Cabante	Camera traps, unhabituated	2016–2022 (6)	1 (0.17)	0	This study
Nimba Mountains, Guinea	Tongbongbon	Camera traps, unhabituated	2022–2023 (2)	3 (1.5)	3 (1.5)	This study
Budongo‐Bugoma Corridor, Uganda	Kihomboza	Camera traps, unhabituated	2022 (1)	1 (1)	1 (1)	This study
Issa Valley, Tanzania	Issa^b^	Camera traps, unhabituated/habituated	2011–2024 (13)	1 (0.08)	0	This study
Issa Valley, Tanzania	Other (non‐Issa)	Camera traps, unhabituated	2011–2024 (13)	1 (0.08)	1 (0.08)	This study

^a^ Research period includes up until the year when the *K* community became extinct (Nakamura et al. 2015).

^b^ ICC behavior at Issa was never observed directly after habituation in 2018.

### 3.2 Demographic, Behavioural and Ecological Data on ICC Cases

Party sizes in ICC camera trap events ranged from one to 22 independent individuals (Table 4), with a median of 6 individuals across all independent events. Demographic composition varied across and within ICC cases. Across ICC cases, an average of 2.3 adult females were detected for every adult male (range 0.5–8.0). We recorded a single interactor with the dead infant in eight out of 10 cases. Nine cases involved one carrier only, while one case involved two different adult females. Nine cases involved adult female carriers, including two young adults, and one involved a juvenile carrier. In two cases (ICC_TB_1, ICC_TB_2), the adult females carrying the dead infants were previously detected carrying their infants alive.

**TABLE 4:** Demographic, behavioural and ecological data associated with ICC cases derived from camera trap surveys.

Site	Community	Case ID	Duration, season	Sex & age of carrier	Age of dead infant (years)	Corpse status	Carrying mode	Reproductive cycle signs of carrier	Alone or in group	Party size	Adult sex ratio (m/f)	Habitat, group activity and/or behaviour	Other behaviours
CNP	Caiquene–Cadique	ICC_CC_1	7 days, transition from dry to wet	Adult female	1–1.5	(a) Fresh, possible injury on lower leg; (b) Mummified	(a) Left arm supporting infant's back; (b) Left arm supporting infant's hips	(a) Not visible; (b) Maximally swollen	Group	(a) 10; (b) 19	(a) 6/3; (b) 5/10	Cashew orchard, travelling	
CNP	Caiquene–Cadique	ICC_CC_2	28 days, dry	(a) Young adult female; (b) Adult female (different individual)	0.5–1	(a) Fresh; (b) Mummified	(a) Right arm supporting infants back and then head; (b) carrying infant with mouth	(a) Enlarged breasts no swelling; (b) Not visible	Group	(a) 8; (b) 10	(a) 0/6; (b) 3/7	(a) Orange orchard, foraging on orange; (b) Cashew orchard, travelling and feeding on cashew	
CNP	Caghode–Cabante	ICC_CGH_1	1 day, dry	Juvenile, ~5 years old	0.5–1.5	Fresh	Dragging infant with right hand by infant's foot/ankle	NA	Group	9	1/8	Old growth forest, travelling	Juvenile carrier display is followed by an adult female who displays enlarged breasts with no anogenital swelling
CNP	Lautchande	ICC_LA_1	2 days, dry	Adult female	0.5–1	Fresh	(a) Switch right arm and left arm supporting infant's back; (b) Right arm supporting infant's neck	(a) No swelling; (b) No swelling	(a) Alone; (b) Group	(a) 1; (b) 3	(a) 0/1; (b) 1/2	(a) Old growth forest, descending from a tree; (b) Forest edge near road, travelling	
NM	Tongbongbon	ICC_TB_1	9 days, dry	(a) Adult female, (b) Adult female (same), (c) Adult female (same)	1–1.5	(a) Fresh; (b) Fresh, but has lost most hair; (c) Mummified	(a) Switch left arm and right arm supporting infants back; (b) Supporting infants back with right arm; (c) Supporting infants back with right arm	(a) Enlarged breasts, no swelling; (b) No swelling; (c) No swelling	(a) Group; (b) Group; (c) Group	(a) 22; (b) 9; (c) 17	(a) 11/8; (b) 5/3; (c) 8/6	(a) Primary forest, travelling; (b) Primary forest, travelling; (c) Primary forest, travelling	(a) Juvenile offspring of adult female carrier looks closely at dead infant as carrier sits for a moment holding the dead infant; (b) ICC carrier is travelling with a young adult female who carries a living infant, observed dead 7 days later and still carried by the same young adult female (ICC_TB_2); (c) Raining
NM	Tongbongbon	ICC_TB_2	5 days, dry	(a) Young adult female, (b) Young adult female (same)	0.5–1	(a) Fresh, but has lost most hair; (b) Mummified	(a) Infant carried on the back with the feet facing forward hanging down over female's shoulders; (b) Infant carried on the back with the feet facing forward hanging down over female's shoulders	(a) Partially swollen; (b) Partially swollen	(a) Group; (b) Group	(a) 16; (b) 20	(a) 7/7; (b) 8/10	(a) Primary forest, travelling; (b) Primary forest, resting	(a) One of the adult females in the party is the carrier from ICC_TB_1, now without the dead infant. Fight between adult males; (b) Large party resting for > 1 h
NM	Tongbongbon	ICC_TB_3	15 days, transition from dry to wet	(a) Adult female, (b) Adult female (same)	1–1.5	(a) Mummified; (b) Mummified	(a) Supporting infants body with left arm; (b) Carrying infant in mouth	(a) No swelling; (b) Partially swollen	(a) Group; (b) Group	(a) 4; (b) 12	(a) 3/1; (b) 6/4	(a) Primary forest, travelling; (b) Primary forest, travelling	(a) Female together with juvenile offspring; (b) Female together with juvenile offspring.
B‐BC	Kihomboza	ICC_KB_1	26 days, dry	Adult female, 35–40 years old	1–1.5	Mummified	Held in one hand in front of her (23 out of 27 events); tucked in her groin pocket when also carrying crop foods (cocoa and jackfruit, 2 events); draped or balanced on her shoulders or mid‐back (8 events)	Day 1 = no swelling. By Day 13 = partial swelling. By Day 24 = maximal swelling. (By Day 18 the female was always closely escorted by adult males)	Group	Average 5.8, range 2–11 across all 27 events	0/5–3/1	All 27 events captured in 2 exotic eucalyptus and agroforestry plantations including 1 mixed with cocoa and coffee trees. All recordings involved travel. Two events included crop feeding.	In one event carrier seemed to drop the dead infant (when preventing her juvenile from climbing on her back) and, when retrieving the corpse from the ground, she seemed to mildly threaten a nearby subadult female. Other community members never showed obvious interest in the corpse in any recording
IV	Issa	ICC_IV_1	1 day, wet season	Adult female	0–0.5	Fresh	(a) slung on carrier's back; (b) Tucked in groin pocket, held in hand, and slung on carrier's back, possible umbilical cord around carrier's body also secures infant	No swelling	Group	2	0/1	Primary forest, (a) travelling; (b) foraging	(a) Mother travelling with juvenile; (b) Mother and juvenile termite fishing
IV	Other (non‐Issa)	ICC_IV_2	1 day, transition from dry to wet	Adult female	2–3	Mummified	Slung on carrier's back	No swelling	Group	3	0/3	Primary forest, travelling	Mother travelling with two other females with infants dorsal

*Note:* Letters in brackets (a, b, c) refer to separate camera events.

The age brackets of dead infants across all ICC cases ranged from 0–0.5 years to 2–3 years, with a median of 1–1.5 years. All seven extended cases involved a dead infant older than 0.5 years, with a median age bracket of 1–1.5 years (ranging from 0.5–1 to 2–3 years).

**FIGURE 2:**
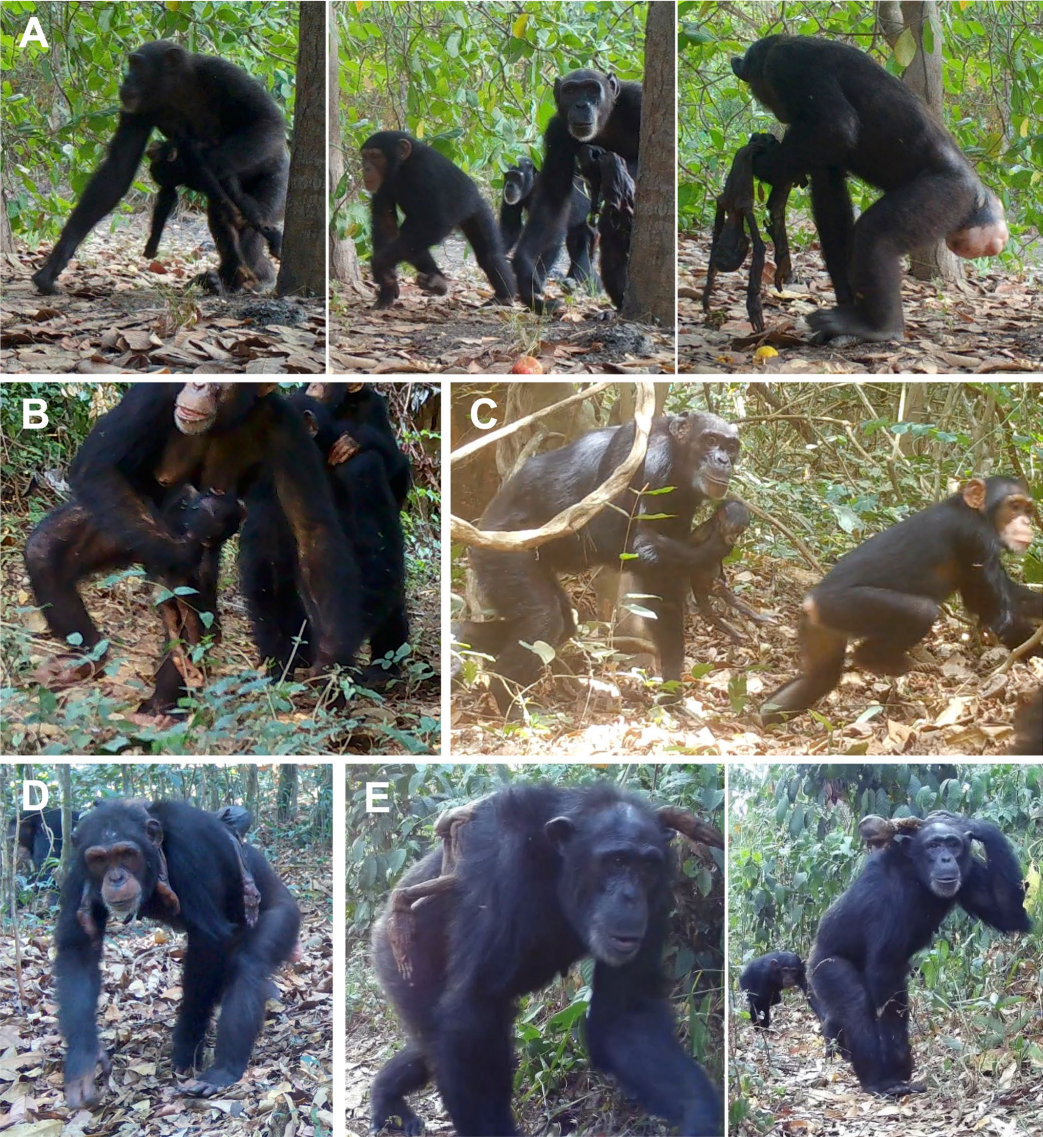
Evidence of ICC behaviour in unhabituated chimpanzees detected by camera traps. (A) The first recorded ICC case in Caiquene–Cadique showing an adult female carrying the fresh carcass (left) and then again, the mummified carcass 7 days later, while maximally swollen (centre and right); (B) young adult female of the Caiquene–Cadique community carrying the corpse of an infant while foraging on oranges; (C–E) atypical carrying modes by adult females in the communities of Lautchande (C), Tongbongon (D) and Kihomboza (E), respectively.

In all ICC cases, the reproductive (anogenital swelling) status of the adult female carrier was visible in camera trap footage, and in two cases, changes in swelling were observed over time. We recorded adult female carriers with maximal swelling (*N* cases = 2, Figure 2A), partial swelling (*N* = 3), no swelling (*N* = 6), and enlarged breasts with no swelling (*N* = 2, Figure 2B). In seven cases, the first camera trap independent events showed the adult female carrier with no swelling. In the case involving a juvenile dragging a fresh carcass (ICC_CGH_1), the adult female following the juvenile displayed enlarged breasts and no anogenital swelling. In one case (Kihomboza, ICC_KB_1), repeated camera trap detections over the 26‐day ICC duration showed that the adult female carrier changed from having no swelling to partial swelling (by day 13) to maximal swelling (by day 24). By day 18, this female was always detected in close company of adult males.

In five cases, typical carrying modes were recorded (i.e., using hands or arms to support the infant's back). Eight cases involved some atypical carrying, including dragging the corpse on the ground, carrying the corpse draped over the shoulders or back, carrying the corpse in the mouth or tucked in the groin, and carrying the corpse by its head or neck (e.g., Figure 2C–E). Three ICC cases also involved the adult female carrier travelling, feeding and/or foraging in agricultural areas, including foraging in an orange orchard within a village (ICC_CC_2, Figure 2B), transporting jackfruit and feeding on a cocoa pod while simultaneously carrying the corpse (ICC_KB_1).

## 4 Discussion

### 4.1 Frequency of Detecting ICC Cases

Most ICC cases in primates are recorded via direct observations at sites where animals are habituated (Biro et al. 2010; Fashing et al. 2011; Das et al. 2019; Lonsdorf, Wilson, et al. 2020; Watts 2020; Fernández‐Fueyo et al. 2021; Soldati et al. 2022). It could be assumed that detection rates of ICC cases are thus higher at sites where chimpanzees are intensively followed. We compared detection rates per year of research and found no evidence that remote camera traps detected ICC cases at a lower rate than direct observations of habituated chimpanzees. In our study, communities where ICC behaviour was recorded, camera traps detected ICC cases 2.3 times more frequently than direct observations, and for the extended ICC cases, camera trap detection rates were nearly eight times higher than long‐term direct observations. In chimpanzees, fission‐fusion dynamics mean that not all individuals can be observed continuously during daily follows. However, extended ICC cases are unlikely to remain undetected in continuously monitored habituated communities. Nonetheless, in some communities (e.g., Issa Valley), adult females are sometimes not seen for months at a time, even if the rest of the community is regularly observed (Fiona Stewart, unpubl. data). It is also possible that ICC rates are actually higher in some of our study communities (Tongbongbon, NM, and Kihomboza, B‐BC, average 1.25 cases and extended cases/year of study) compared to the other communities in our study (average 0.14 cases/year, 0.06 extended cases/year across the five other unhabituated communities), which are more similar to those reported in the literature. Ideally, comparative camera trap‐based behavioural studies should consider trapping effort (measured in camera trap days) for each community, especially when studying more commonly detected behaviours. We did not provide this information here, as the data were unavailable for some of our sites and study periods. While we acknowledge this limitation, we note that our aim was to compare yearly ICC detection rates with those from direct observations, meaning that a finer temporal resolution of effort would not have added significant additional insight.

Another important consideration is the higher proportion of extended ICC cases (and a higher median duration across all cases) recorded by camera traps compared to available data from direct observations (e.g., the median carrying duration of our cases was 7 days, compared to 2 days at Gombe, Lonsdorf, Wilson, et al. 2020). This suggests that camera traps may be more effective at detecting extended cases rather than short ICC instances. Indeed, neither direct observations nor camera traps can guarantee the detection of all cases. However, unlike human observers, camera traps can monitor a larger area over extended periods, enabling the simultaneous observation of more individuals both within and across communities. This may increase the likelihood of detecting extended cases compared to data collection through direct follows of habituated communities, which may be limited by logistical constraints such as limited or lost visual contact and the need for breaks.

All but one of the 44 independent camera trap events of ICC captured carriers in social groups (parties) rather than alone or with dependents only. This suggests that carriers do not isolate themselves from their social groups, making it unlikely that the higher detection of extended cases in our study results from camera traps being more effective at capturing potentially elusive, lone females displaying ICC than direct observations. It is also possible that the behaviour is underreported and that ICC cases from published findings need to be updated. However, many of the published sources we used collated long‐term data (e.g., Bossou, Guinea: Biro et al. 2010; and Gombe, Tanzania: Lonsdorf, Wilson, et al. 2020), making this unlikely. Our findings demonstrate that camera traps are a powerful tool to detect infrequent wildlife behaviours that are impossible to experimentally produce, such as ICC.

### 4.2 Duration of ICC

Aside from detection rates, we were able to extract information on the condition of the carcass from camera trap footage, which showed it either fresh or fully mummified, and in four of 10 cases, progressing from fresh to mummified. The condition of the carcass and repeated camera trap events allowed us to estimate the minimum duration of ICC. We considered ICC as extended when the corpse appeared mummified, regardless of the time between the first and last camera trap events. In two cases (ICC_CC_1 and ICC_TB_1), the carcass appeared fresh with hair at first detection and mummified after six and nine days only. A limitation of camera traps is the lack of consistent detections over short periods, shown by the low average number of independent events per ICC case (median = 2). It is possible that in ICC cases with detection intervals less than 10 days, but where the carcass appeared mummified (*N* = 4), the carrying duration was indeed less than 10 days. Obtaining more camera trap footage at finer timeframes would allow for a more detailed observation of the decay process. Achieving this may require deploying additional camera traps to increase detections, which can be costly, and detection is not guaranteed. However, the relatively large sample of recordings from the Kihomboza case (ICC_KB_1; *N* = 27 independent events obtained over 26 days, recorded at two locations only) shows that camera traps deployed at habitually used locations can generate significant footage of rare behaviours, particularly when using video mode, which can increase the detection of different individuals in the (travelling) party.

Our data suggest that slow decomposition during drier months may impact ICC duration. We detected only one case in the rainy season, involving a fresh carcass, and two cases during the dry‐rainy seasonal transition period, during which the carcass mummified. At Gombe, nearly two‐thirds of ICC cases were recorded during the dry season, and only two out of 33 were confirmed as extended cases (Lonsdorf, Wilson, et al. 2020). In contrast, at Budongo, over half of the ICC cases, including two that extended over 56 and 89 days, occurred during months with high precipitation (Soldati et al. 2022). Other studies have also found no link between climate and ICC occurrence or duration (Das et al. 2019; Fernández‐Fueyo et al. 2021). Larger species‐specific datasets, including data from camera trap surveys across a wider range of environmental conditions, may help further clarify the extent to which prolonged carrying is facilitated by the environment. The increasing availability of detailed global historical climatic and land cover data at fine spatiotemporal resolutions (e.g., WorldClim, GEDI mission) will facilitate the inclusion of additional, fine‐grained environmental variables, such as monthly precipitation, vegetation cover, and canopy height.

### 4.3 Demographic and Physical Status and Behaviour of Individuals Involved in ICC

In their analysis of 409 ICC cases across 40 primate species, Fernández‐Fueyo et al. (2021) found support for the mother–infant bond hypothesis, showing that the corpses of infants of intermediate age between birth and weaning were carried for longer durations. In the wild, chimpanzees are typically weaned at around 5 years of age (Lonsdorf, Stanton, et al. 2020). From camera trap footage alone, we were not able to determine the precise age of dead infants in months or days, though we estimated an age range of 0–0.5 years to 2–3 years, with extended cases between 0.5–1 and 2–3 years. The age range of our extended cases falls within a range when the mother–infant bond is assumed to be strong, supporting the mother–infant bond hypothesis.

The reproductive stage of an adult female carrier may influence ICC duration. It is hypothesised that the physiological changes associated with pregnancy and birth facilitate continued care of corpses, with abandonment becoming more likely once lactation ends and the reproductive cycle resumes (post‐parturient condition hypothesis, Kaplan 1973). The reproductive cycle of female chimpanzees can sometimes be difficult to infer from camera trap footage alone, for example, due to detection angles or blurry or partial detection, but it is nonetheless often feasible. The swelling stage of the adult female carrier was visible in all our ICC cases. In two extended cases (ICC_CC_1; ICC_KB_1), the female carrier displayed maximal anogenital swelling by the time of the last recorded camera trap evidence, and in two other extended cases (ICC_TB_2; ICC_TB_3), the adult female carrier showed partial swelling. Five other cases showed the female carrier with no swelling, and in two of these (ICC_CC_2; ICC_TB_1), camera trap footage showed an adult female with enlarged breasts carrying the fresh infant carcass, suggesting she was lactating. Our evidence therefore confirms that, in at least four out of nine cases, the adult female carrier resumed the reproductive cycle over the period of ICC. Alongside additional ICC cases, such data, which can be retrieved from camera traps, will be useful for testing the mother–infant bond and post‐parturient condition hypotheses (Kaplan 1973; Watson and Matsuzawa 2018; Lonsdorf, Wilson, et al. 2020; Fernández‐Fueyo et al. 2021).

The age of the carrier is relevant to primate thanatology. It is hypothesised that younger mothers perform more ICC due to their inexperience with motherhood and therefore less awareness that the infant has died (death unawareness hypothesis, e.g., Das et al. 2019). The limitations of camera trap monitoring of unhabituated chimpanzee communities make it difficult to determine the exact ages of individuals unless they have been identified and monitored from birth. Large‐scale camera trap datasets will have coarser demographic variables compared to data from longitudinal observations of habituated individuals. However, age classes (rather than exact ages) can still be informative for addressing demographic questions (McCarthy et al. 2018, 2019; Debetencourt et al. 2023) and can be used in cross‐species comparative thanatology studies (Fernández‐Fueyo et al. 2021). For example, two of the nine ICC cases involving female carriers in our study were identified as ‘young adults’. Thus, camera traps remain a valuable tool, allowing estimation of the approximate ages of carriers and contributing useful data for testing the death awareness hypothesis using maternal age as a predictor (Lonsdorf, Wilson, et al. 2020; Fernández‐Fueyo et al. 2021).

Kinship and the history of the mother, such as whether she had previous offspring (another measure for maternal experience), are much harder to infer in unhabituated communities. Lonsdorf, Wilson, et al. (2020) noted that, in most cases, the dead infant's siblings were the second most likely to interact with the corpse, following the mother. Based on reproductive cycle signs and, in two cases (ICC_TB_1, ICC_TB_2), footage of the adult female carriers with live infants prior to ICC, we assume that all cases except one (ICC_CGH_1) involved the mothers of the deceased infants. In the case involving two adult female carriers (ICC_CC_2), it is likely that the mother was the first carrier, as she showed enlarged breasts. In the single case of a juvenile carrier (ICC_CGH_1), who was followed by an adult female with enlarged breasts, the carrier's age category raises the possibility that s/he was an older sibling of the deceased infant, travelling with the mother. ICC behaviour by non‐mothers has been explored as a potential response to an attraction to or caring impulse for inanimate mammal‐like objects, similar to a maternal response (mammalian‐cues hypothesis, Reiderman et al. 2024). In our case, the rough carrying mode suggests that the behaviour may be more driven by an attraction to a novel object, which is relatively common in juvenile nonhuman primates (Reiderman et al. 2024), rather than a caring response.

None of the independent events, including a party, showed additional individuals interacting with the corpse, except for one instance where a juvenile can be seen closely observing it (ICC_TB_1) and another case involving two distinct carriers (ICC_CC_2). This contrasts with findings from other studies such as Lonsdorf, Wilson, et al. (2020), where most ICC cases involved multiple individuals interacting with the corpse. The low detection of multiple interactors in our sample may be attributed to the fact that most camera trap observations are of short duration and primarily involve travel, often missing instances where individuals sit and interact socially with one another, which would more likely allow for observation of multiple interactors.

Besides demography, relatedness among the corpse interactors, and the mother's experience, other contextual factors, such as the cause of death and how individuals handle the corpse, can also inform the death awareness hypothesis. Among primates, non‐traumatic deaths, such as those caused by illness, might lead to a higher chance of ICC because the sensory cues associated with death are less apparent (death awareness hypothesis, e.g., Anderson 2011; Das et al. 2019; Gonçalves and Carvalho 2019; Lonsdorf, Wilson, et al. 2020; Fernández‐Fueyo et al. 2021). However, it is largely impossible to determine the cause of death of the infant from camera trap data alone. Despite this limitation, camera traps revealed data on carrying modes, which can offer insights into death awareness (Lonsdorf, Wilson, et al. 2020). Handling and carrying behaviours that differ from those shown towards living infants suggest an ability to discriminate between living and non‐living states (Gonçalves and Carvalho 2019; Lonsdorf, Wilson, et al. 2020). Eight out of our 10 cases involved atypical transport. Of the seven ICC cases where footage of a fresh carcass was available, five involved atypical transport. Our data are consistent with previous literature showing that atypical transport in ICC by chimpanzees is common and tends to occur shortly after the infant's death (Lonsdorf, Wilson, et al. 2020).

## 5 Conclusions

Our study demonstrates the potential of camera traps for studying infrequent and unpredictable behaviours, such as ICC in unhabituated wild chimpanzees. Camera traps detected ICC cases at higher rates compared to direct observations in habituated communities, making them useful in recording behaviours that would otherwise be virtually impossible to observe in unhabituated communities. Our study illustrates how camera traps can help to identify factors influencing ICC behaviour, although it remains a challenge to determine exact ages, kinship, or causes of death from camera trap footage alone. In addition to associated environmental data (informing the slow decomposition hypothesis), camera traps can capture the approximate age of the infant (informing the mother–infant bond hypothesis) and the reproductive cycle of the adult female carrier (informing the post‐parturient condition hypothesis), as well as the approximate age of the adult female carrier and carrying modes (informing death awareness hypotheses). By expanding the scope of data collection across various environmental conditions and across multiple (unhabituated and habituated) communities, camera traps offer the potential to enhance our understanding of primate thanatology and the evolutionary underpinnings of animal cognition and emotions related to death. This study supports the use of remote camera traps not only to monitor wildlife population trends but also aspects of their ecology, behaviour, and health. Camera traps might facilitate a switch from the opportunistic recording of rare behaviours to a more systematic approach possibly facilitated by the use of AI tools for data processing.

## Author Contributions

**Elena Bersacola:** conceptualization (equal), data curation (equal), formal analysis (lead), funding acquisition (equal), investigation (equal), methodology (equal), project administration (equal), resources (equal), validation (equal), visualization (lead), writing – original draft (lead), writing – review and editing (equal). **Matthew R. McLennan:** conceptualization (equal), data curation (equal), formal analysis (equal), funding acquisition (equal), investigation (equal), methodology (equal), project administration (equal), resources (equal), validation (equal), writing – original draft (supporting), writing – review and editing (equal). **Joana H. Bessa:** data curation (equal), funding acquisition (supporting), investigation (equal), methodology (supporting), project administration (supporting), resources (equal), validation (supporting), writing – original draft (supporting), writing – review and editing (equal). **Henry Camara:** data curation (equal), investigation (equal), methodology (supporting), project administration (supporting), resources (supporting), validation (supporting), writing – review and editing (supporting). **Maimuna Jaló:** data curation (equal), investigation (equal), methodology (supporting), project administration (supporting), resources (supporting), validation (supporting), writing – review and editing (supporting). **Vicent Kiiza:** data curation (equal), investigation (equal), methodology (supporting), project administration (supporting), resources (supporting), validation (supporting), writing – review and editing (supporting). **Gnan Mamy:** data curation (equal), investigation (equal), methodology (supporting), project administration (supporting), resources (supporting), validation (supporting), writing – review and editing (supporting). **Nicholas Mpanga:** data curation (equal), investigation (equal), methodology (supporting), project administration (supporting), resources (supporting), validation (supporting), writing – review and editing (supporting). **Vicky Oelze:** data curation (equal), investigation (equal), methodology (supporting), project administration (supporting), resources (supporting), validation (supporting), writing – review and editing (supporting). **Marina Ramon:** data curation (equal), formal analysis (supporting), investigation (equal), methodology (equal), project administration (supporting), resources (equal), validation (supporting), writing – original draft (supporting), writing – review and editing (equal). **Américo N. W. Sanhá:** data curation (equal), investigation (equal), methodology (supporting), project administration (supporting), resources (supporting), validation (supporting), writing – review and editing (supporting). **Laura van Holstein:** data curation (equal), investigation (equal), methodology (supporting), project administration (supporting), resources (supporting), validation (supporting), writing – review and editing (supporting). **Maegan Fitzgerald:** data curation (equal), formal analysis (supporting), funding acquisition (equal), investigation (equal), methodology (equal), project administration (equal), resources (equal), validation (equal), writing – review and editing (supporting). **Fiona A. Stewart:** conceptualization (supporting), data curation (equal), formal analysis (supporting), funding acquisition (equal), investigation (equal), methodology (equal), project administration (equal), resources (equal), validation (equal), writing – review and editing (equal). **Kathelijne Koops:** conceptualization (equal), data curation (equal), formal analysis (supporting), funding acquisition (equal), investigation (equal), methodology (equal), project administration (equal), resources (equal), validation (equal), writing – review and editing (equal). **Kimberley J. Hockings:** conceptualization (lead), data curation (equal), formal analysis (equal), funding acquisition (equal), investigation (equal), methodology (equal), project administration (equal), resources (equal), supervision (equal), validation (equal), writing – original draft (equal), writing – review and editing (equal).

## Conflicts of Interest

The authors declare no conflicts of interest.

## Data Availability

Camera trap data containing ICC can be accessed from: https://zenodo.org/records/13374119.
